# Global research trends of myopia in children and adolescents: A bibliometric review with hotspot visualization

**DOI:** 10.1097/MD.0000000000050124

**Published:** 2026-08-07

**Authors:** Xiao-Lei Yin, Shu-Xing Ji, Xiu-Xin Li

**Affiliations:** a Department of Ophthalmology, The 305 Hospital of PLA, Beijing, China; b Department of Ophthalmology, Daping Hospital, Army Medical University, Chongqing, China.

**Keywords:** bibliometrics analysis, children, global trends, myopia, VOSviewer

## Abstract

**Background:**

The rapidly rising global prevalence of childhood myopia has become a major public health concern, accompanied by a continuous annual increase in relevant academic publications. Against this backdrop, this study adopted bibliometric methods to analyze the research hotspots and evolutionary trends in this field.

**Methods:**

Publications related to myopia in children and adolescents were collected from the Science Citation Index Expanded of the Web of Science Core Collection database. A bibliometric and visualization analysis was performed using Microsoft Office Excel and VOSviewer to identify the most influential countries, papers, and authors and to construct the visualization map of citation, co-citation, and co-occurrence.

**Results:**

After screening, 3413 papers met the inclusion criteria. China had the largest number of published papers, whereas the United States enjoyed the highest citations. Moreover, cooperation network analysis showed close cooperation among China, the United States, and Australia. The review “Myopia” in 2012 was the most-cited article. Among the 10 most-cited articles, outdoor time and the development of myopia in children were the topics with the highest frequency. As an expert in the field of epidemiology of myopia research, Saw SM contributed the most citations. The burst keywords were divided into 3 clusters through co-occurrence analysis: epidemiology, management, and mechanism of myopia in children and adolescents.

**Conclusion:**

This bibliometric analysis of myopia in children and adolescents helps researchers identify hotspots and research frontiers in the field and might provide a direction that future research should concentrate on.

## 1. Introduction

Myopia, also known as near-sightedness or short-sightedness, is the most common refractive error that has increased in prevalence globally and is projected to affect nearly half of the population of the world by 2050.^[1]^ The increasing prevalence of myopia during early childhood has heightened the risk of developing high myopia. High myopia is associated with various specific complications, including cataracts, glaucoma, retinal detachment, and macular degeneration, which may lead to vision impairment and blindness.^[2]^ The rapid increase in the prevalence of myopia among children worldwide is regarded as a major public health concern.

The age of myopia onset has been observed to decrease in recent years, which may be attributed to modifiable risk factors such as increased intensive education, increased near-work activities, excessive use of electronic products, and frequent consumption of sweets.^[3]^ To date, a variety of interventions have been proposed to slow the progression of myopia, including low-dose atropine eye drops, contact lenses, an increase in outdoor time, and orthokeratology. Given the expanding body of research on myopia in children and adolescents, it is essential to assess the scientific output and trends in this field.

Bibliometric analysis is a quantitative approach that employs mathematical and statistical techniques, combined with visualization tools, to reveal numerous aspects and trends in the evolution of a scientific subject.^[4]^ To our knowledge, no comprehensive bibliometric studies have explored myopia in children and adolescents. Hence, the aim of this study is to systematically analyze the research on myopia in children and adolescents and to evaluate hotspot research and future trends, further guiding clinical and basic research.

## 2. Materials and methods

**Figure 1.**
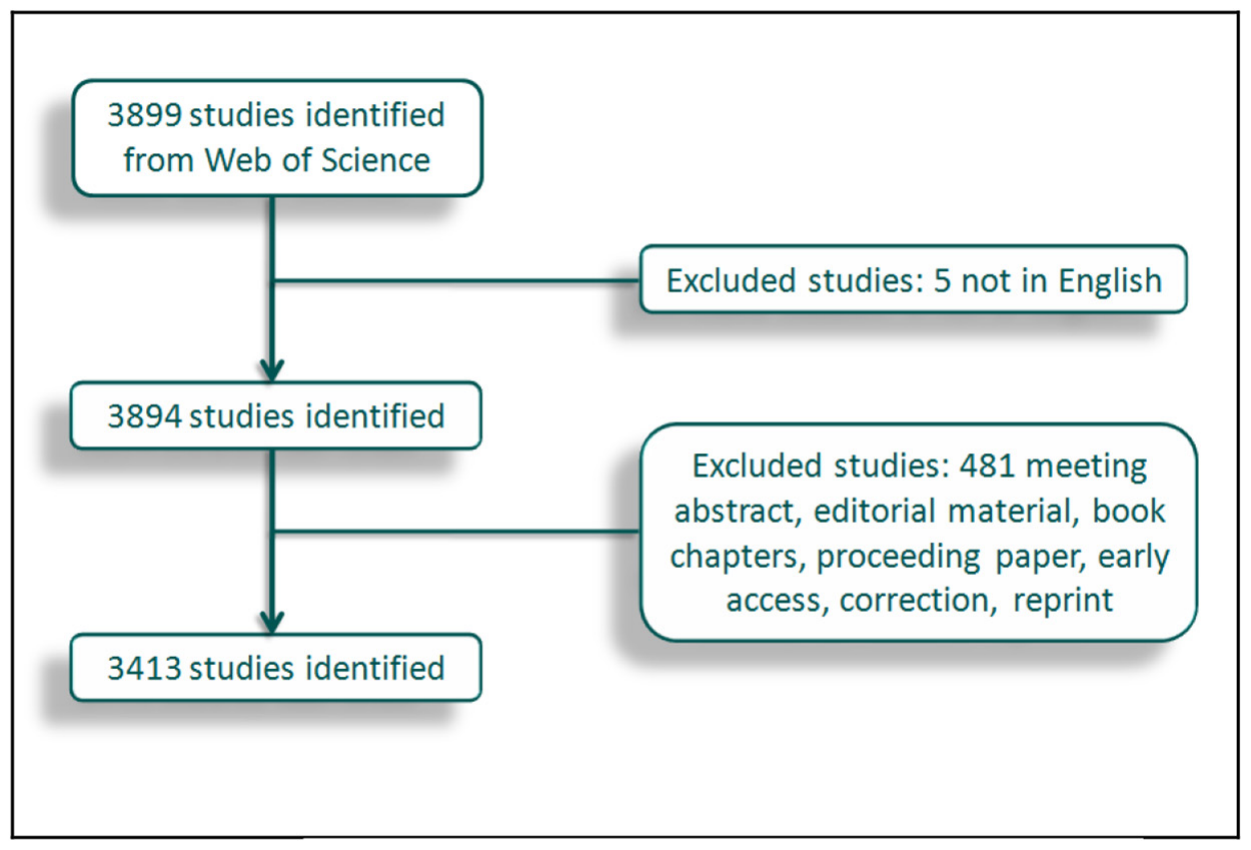
Flow chart of literature retrieval research.

We performed a systematic search in the Science Citation Index Expanded of Web of Science Core Collection (WoSCC). The search terms included: (TI = (“child*” OR “adolescent*” OR “juvenile” OR “pediatric*”) OR AB = (“child*” OR “adolescent*” OR “juvenile” OR “pediatric*”)) AND (TI = (“myopia” OR “shortsightedness” OR “nearsightedness”) OR AB = (“myopia” OR “shortsightedness” OR “nearsightedness”)). Only articles and reviews written in English were included from 1900 to 2025 (retrieved on January 6, 2025). Finally, 3413 papers meeting the criteria for inclusion were obtained. All valid documents retrieved from WoSCC were prepared in a format suitable for Microsoft Office Excel 2019 and VOSviewer (version 1.6.20). We determined the most influential countries, papers, and authors and constructed the visualization map of citation, co-citation, and co-occurrence. The details of the search strategy and literature filtering are presented in Figure 1.

This bibliometric study adopted mathematical and statistical analytical approaches. As no human or animal subjects were involved in this work, ethical approval was not required in accordance with national regulations and institutional guidelines.

## 3. Results

### 3.1. Annual publications and citations

**Figure 2.**
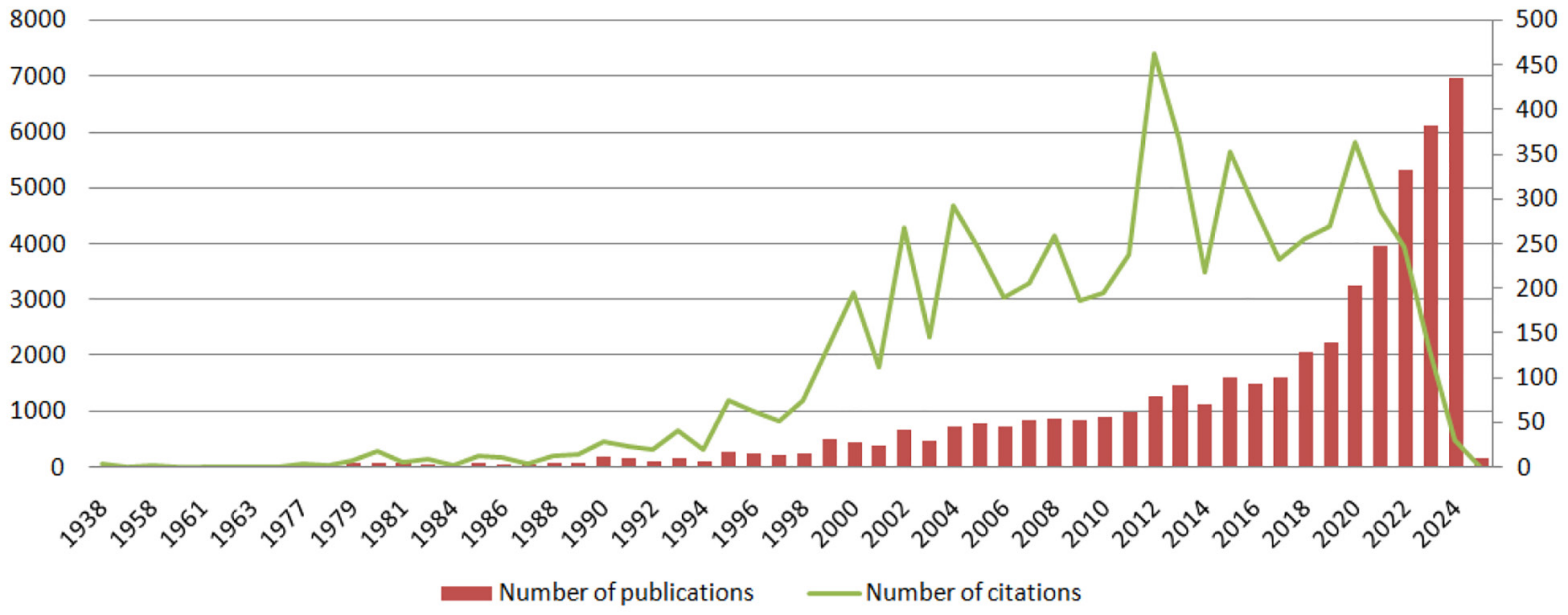
The annual publication and citations number of publications.

The first article on myopia in children and adolescents was published in 1938. Before 2002, this research area received little attention. By 2007, the number of published articles exceeded 50. The quantity of publications increased rapidly from 91 in 2013 to 436 in 2024. The number of citations had exceeded 1000 in 1995, peaking at 7419 in 2012. The substantial increase in publications and citations after 2012 indicated growing research interest in this field (Fig. 2).

### 3.2. Bibliometric analysis of the collaboration network

**Figure 3.**
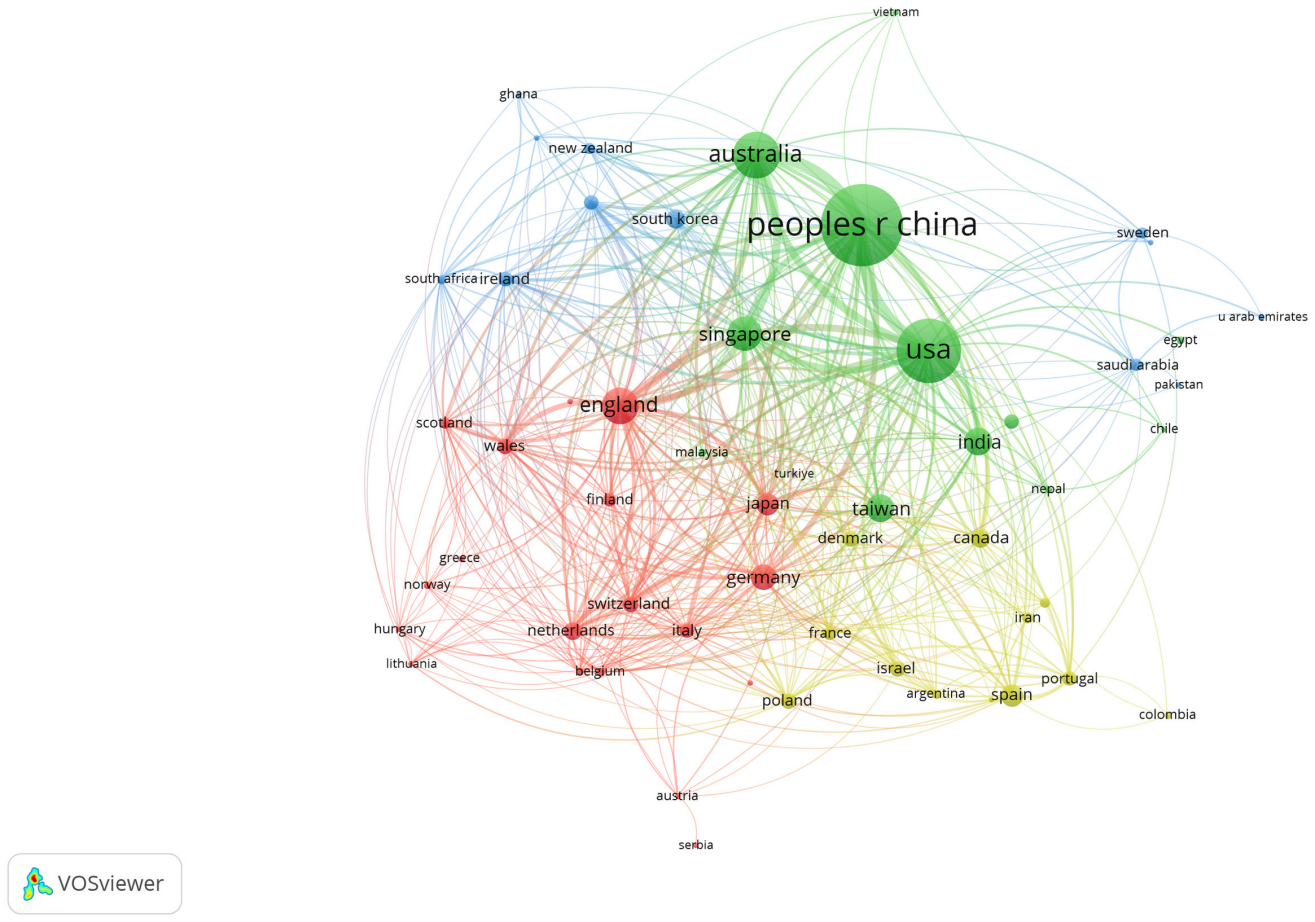
VOSviewer network visualization of research collaborations among countries.

A total of 56 countries and regions contributed to the research on myopia in children and adolescents. Figure 3 presents a collaborative network map for each country or region, and larger nodes indicate that more publications have been contributed by the corresponding country. The most productive country/region is the People’s Republic of China (1294), followed by the United States (789), Australia (421), England (258), and Singapore (230). However, the United States enjoyed the highest citations of 39,401, and the People’s Republic of China followed with 31,614 (Table 1). Connections among nodes signify cooperative efforts among countries or regions, with more connections indicating closer collaboration. According to the map, the People’s Republic of China has cooperation with many countries, among which the 2 most important are the United States and Australia. This discovery suggests that these countries may have played a crucial role in the research in this field.

**Table 1:** Top 10 most influential countries on myopia in children and adolescents.

Rank	Country/region	Number of publications	Number of citations
1	China	1294	31,614
2	USA	789	39,401
3	Australia	421	22,609
4	England	258	12,497
5	Singapore	230	17,031
6	India	156	3121
7	Taiwan, China	149	6162
8	Germany	126	4533
9	Spain	97	2227
10	Japan	96	5705

### 3.3. High-impact publications

**Figure 4.**
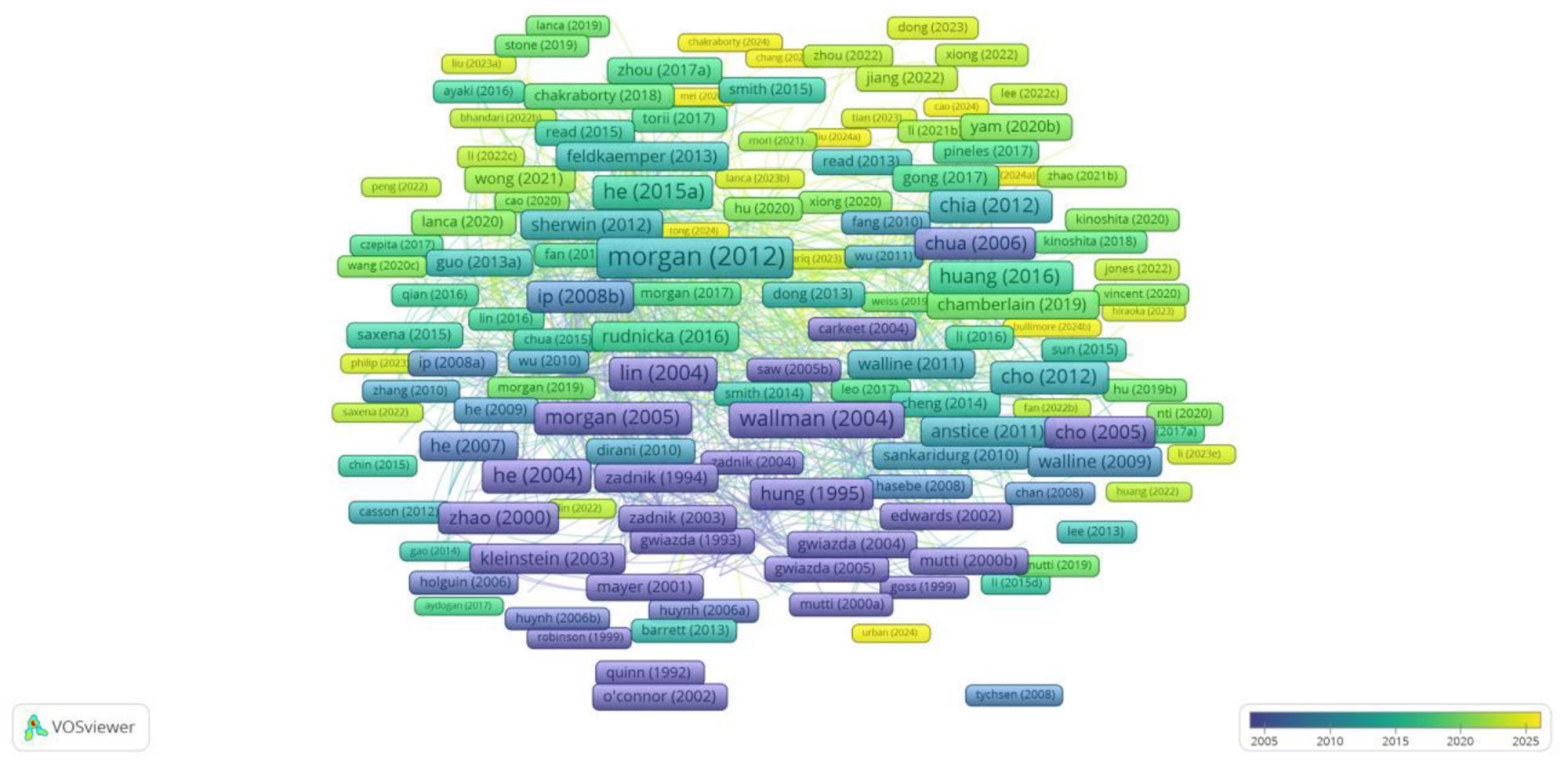
Overlay visualization of the publications.

Figure 4 shows the most-cited publications. The colorful circles each correspond to a cited article. The size of the circle represents the article’s citation weight. High-impact publications with high cited counts indicate well-regarded findings and hotspots of research in this field.

Among the 10 most-cited articles (Table 2), there are 7 research papers and 3 review articles. Of the top 4 most-cited articles, one stands out as a review that delves into the critical role of visual cues in the growth of the eyes,^[5]^ while the other 3 are dedicated to exploring the correlation between outdoor time and the development of myopia in children.^[6–8]^ These studies suggest that increased time spent outdoors is inversely associated with the incidence of myopia. Genetic factors play a paramount role in the onset of myopia among adolescents. There is a notable familial aggregation: myopic children often have parents with the same condition.^[9]^ In addition, observations show that children with the highest levels of outdoor activity and non-myopic parents exhibit the lowest risk of developing myopia compared with those with 2 myopic parents.^[10]^ The widespread prevalence of myopia-related visual impairment has led to a considerable number of children being unnecessarily prescribed corrective spectacles.^[11]^ Indeed, to reduce the prevalence and severity of myopia, more attention should be paid to eye care for preschool-aged children.^[12]^ The use of orthokeratology lenses has been demonstrated to be an effective method for controlling the progression of myopia. Compared with subjects wearing single-vision glasses, those wearing orthokeratology lenses exhibit a slower increase in axial elongation.^[13]^ In addition, population-based research on children in China indicates a higher prevalence of myopia in urban settings and among ethnic minorities. Environmental factors also play a crucial role in the development of myopia.^[14]^

**Table 2:** Top 10 cited publications in myopia among children and adolescents citation network.

Rank	Title	Author	Year	Document type	Number of citations
1	Myopia	Morgan IG	2012	Review	1328
2	Outdoor activity reduces the prevalence of myopia in children	Rose KA	2008	Article	864
3	Homeostasis of eye growth and the question of myopia	Wallman J	2004	Review	770
4	Effect of time spent outdoors at school on the development of myopia among children in China: a randomized clinical trial	He MG	2015	Article	656
5	Prevalence of myopia in Taiwanese schoolchildren: 1983 to 2000	Lin LL	2004	Article	645
6	Refractive error and visual impairment in urban children in southern China	He MG	2004	Article	604
7	Worldwide prevalence and risk factors for myopia	Pan CW	2012	Review	592
8	Parental history of myopia, sports and outdoor activities, and future myopia	Jones LA	2007	Article	543
9	Parental myopia, near work, school achievement, and children’s refractive error	Mutti DO	2002	Article	534
10	Retardation of myopia in Orthokeratology (ROMIO) study: a 2-year randomized clinical trial	Cho P	2012	Article	531

### 3.4. Most influential authors

**Figure 5.**
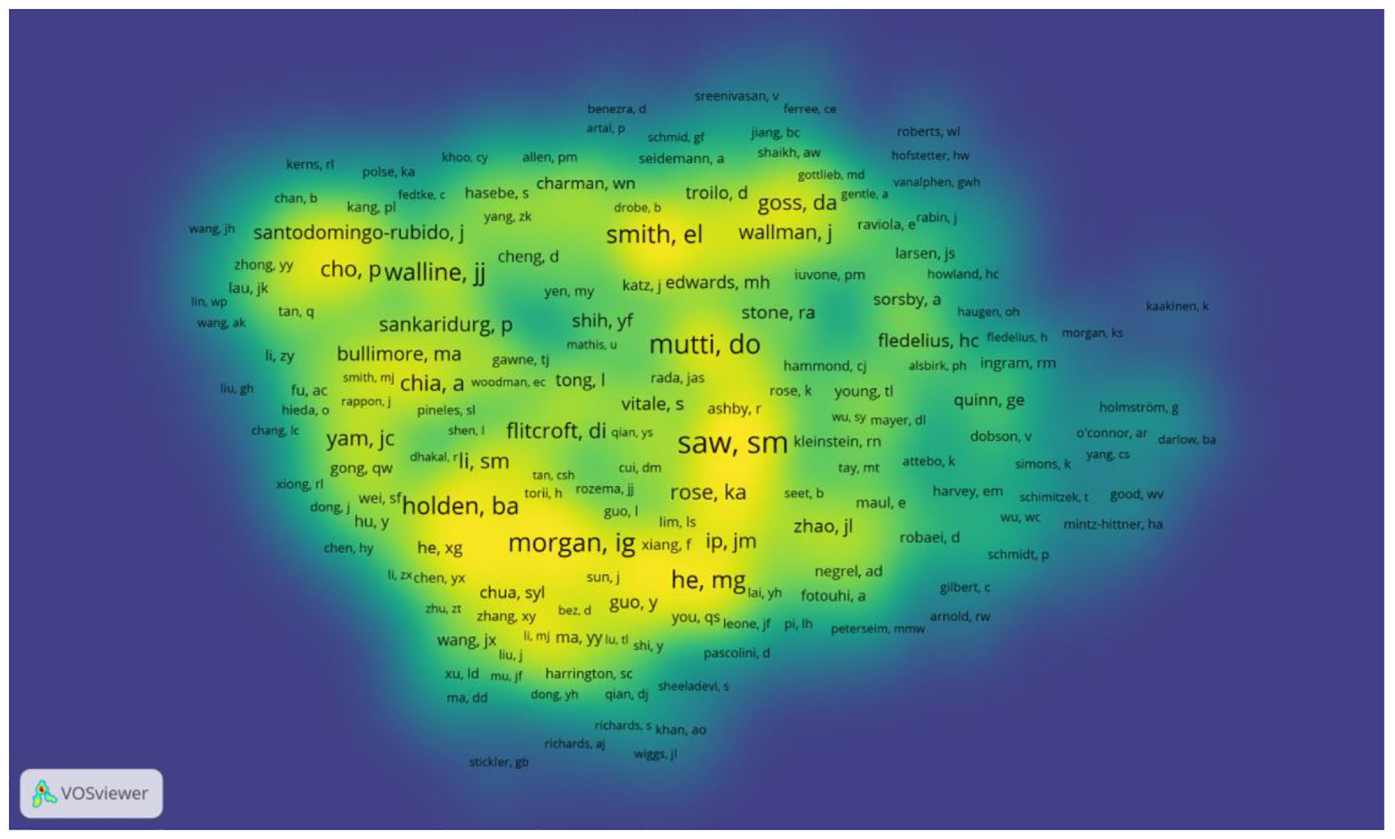
Density visualization for authors in co-citation network map.

Author co-citation analysis was introduced in 1981. It reflects that an author is influenced by the work of another author and has been used as an effective method for identifying the intellectual structure of a research domain. Through co-citation analysis, the most influential authors are visualized by a density network map. Names of authors in yellow emerge more frequently, while green and blue emerge less frequently (Fig. 5). A total of 28,071 researchers are included in this analysis. Among all authors, Saw SM, an expert in the field of epidemiology of myopia research, contributed the most citations. Two other authors with co-citations over 1000 are Mutti DO and Morgan IG. Morgan IG also focuses on the epidemiology of myopia. Mutti DO’s research centers on juvenile myopia. Smith EL specializes in the area of animal models for visual development. Other well-known scholars in myopia research among children and adolescents include Gwiazda J, Holden BA, Walline JJ, He MG, Zadnik K, and Wu PC. Half of the top 10 most influential authors come from the United States (Table 3).

**Table 3:** Top 10 most influential authors according to the author co-citation analysis.

Rank	Author		Organization	Country/region	Total link strength	Citations
1		Saw SM	National University of Singapore	Singapore	66,863	1805
2		Mutti DO	Ohio State University	USA	52,894	1220
3		Morgan IG	Australian National University	Australia	39,412	1120
4		Smith EL	University of Houston	USA	49,900	916
5		Gwiazda J	New England College of Optometry	USA	41,177	890
6		Holden BA	University of New South Wales	Australia	27,875	882
7		Walline JJ	Ohio State University	USA	42,165	846
8		He MG	Sun Yat-sen University	China	28,949	832
9		Zadnik K	University of California	USA	28,539	729
10		Wu PC	Chang Gung Memorial Hospital	Taiwan, China	28,746	707

### 3.5. Distribution of keywords

**Figure 6.**
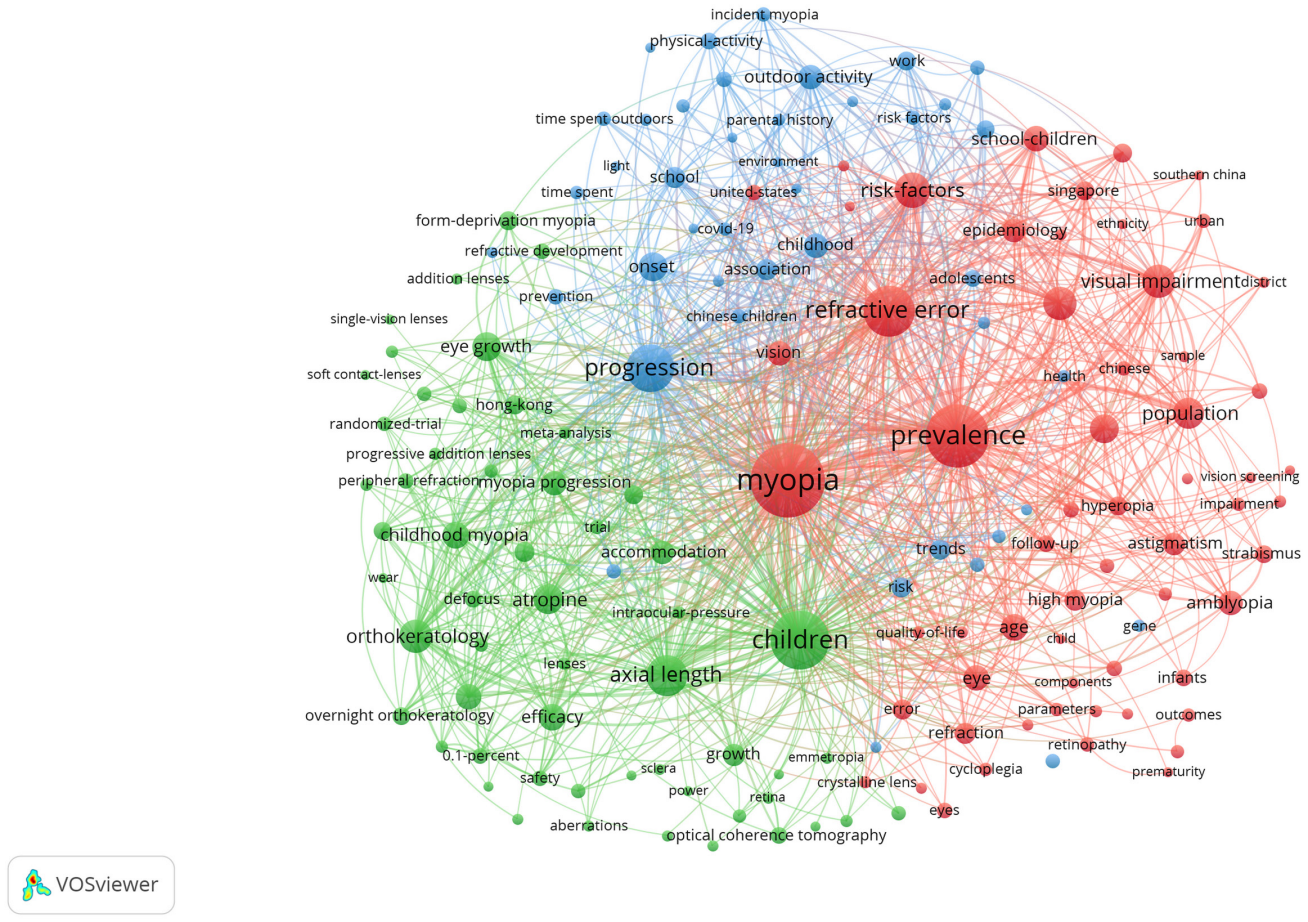
Co-occurrence network of keywords.

Structural analysis of keywords provides insight into the most popular topics in publications in a specific research field. Through the keyword co-occurrence analysis, 159 (the minimum number of occurrences of a keyword was set at 30) of 6620 retrieved keywords from abstracts and titles satisfy the threshold (Fig. 6).

This visualization map identifies 3 different clusters based on similarities, and each cluster summarizes a specific theme. Cluster 1 (red) represents keywords related to the epidemiology of myopia in children and adolescents. Extracted co-occurrence keywords include “myopia,” “prevalence,” “refractive error,” “risk-factors,” “visual impairment,” “schoolchildren,” “population,” “refractive errors,” “age,” and “eye.” Cluster 2 (green) is linked with the management of myopia in children and adolescents. The extracted co-occurrence keywords include “children,” “axial length,” “orthokeratology,” “atropine,” “eye growth,” “childhood myopia,” “efficacy,” “myopia control,” “myopia progression,” and “accommodation.” Cluster 3 (blue) represents keywords about the mechanism of myopia in children and adolescents, including “progression,” “onset,” “outdoor activity,” “childhood,” “school,” “association,” “trends,” “risk,” “work,” and “china.”

## 4. Discussion

The incidence of myopia in children varies greatly across different regions worldwide. Areas with the highest prevalence of myopia among school-aged children include East Asia, Singapore, urban regions of China, Taiwan, and South Korea.^[15]^ In contrast, the prevalence is lower in South America, the Middle East, and Africa. However, there is a rising trend observed in all these regions.^[16]^ Hereditary factors constitute one of the risk factors contributing to the onset of myopia in pediatric populations. Children with myopic parents are more prone to developing myopia compared with children of non-myopic parents.^[17]^ The most significant environmental factor is the amount of time spent outdoors, which has been shown to delay the progression of myopia in children.^[18,19]^ Moreover, near work is also a risk factor for myopia. Research has indicated that this risk is particularly relevant for children at the age of 6, and not for the 12-year-old cohort.^[20]^ This finding implies that near work may act as a precipitant for the earlier onset of myopia in smaller children.^[16]^ In addition, gender differences were also observed, where daily reading sessions exceeding 2 hours were positively associated with myopia in boys, while weekly time spent watching television was linked to myopia in girls.^[21]^ Insufficient sleep duration serves as an independent risk factor for myopia. Specifically, children who sleep <8 hours have a higher risk of developing myopia compared with those who sleep more than 9 hours.^[22]^ Dim light exposure is important for human myopia development, too. Compared with non-myopic children, myopic children exhibit reduced exposure to low-light environments with less stimulation of rod pathways.^[23]^ Furthermore, classic epidemiological evidence strongly suggests a causal relationship between education and myopia. In modern societies, the onset of myopia in the majority of humans occurs during the period of childhood education, whereas children who do not go to school rarely develop myopia.^[24]^

Indeed, most of the current treatments or strategies for managing myopia in children are based on the various hypothesized mechanisms behind the development of myopia. The protective effect of outdoor activity against the onset of myopia is predicated on the hypothesis that exposure to higher light intensities in natural environments during daylight hours leads to the stimulation of a greater secretion of dopamine in the retina, thereby suppressing the axial elongation of the eye, which is a critical determinant in the pathogenesis of myopia.^[7]^ This hypothesis has been supported by animal experiments.^[15]^ The evidence from school-based intervention trials indicates that an increase in daily outdoor time by 40 to 80 minutes can significantly reduce the incidence of myopia. Moreover, a daily allocation of 2 hours to outdoor activities can provide significant protection from myopia.^[15]^ Numerous peer-reviewed studies have shown that wearing orthokeratology lenses overnight can effectively slow the progression of myopia in children.^[25]^ However, the use of orthokeratology contact lenses to compress or manipulate corneal curvature is not based on the understanding of the pathogenesis. In the 1950s, it was noted that individuals experienced an improvement in visual acuity and a reduction in myopic correction following the removal of rigid contact lenses. Based on this, OrthoK lenses were developed.^[26]^ A recent meta-analysis reported that OrthoK has the ability to reduce axial length elongation by approximately 50% after monitoring for 2 years. Although data suggest that the efficacy of OrthoK diminishes over time, this reduction may not be due to a decrease in the effectiveness of OrthoK itself, but rather to a natural slowing of myopia progression in the control group with age.^[13]^ Compared with other strategies, pharmacological intervention is the most effective method for controlling myopia.^[27]^ At present, atropine is the available anticholinergic drug in myopia treatment with demonstrated consistent efficacy, which can block the action of acetylcholine in the developing retina, thereby regulating the growth of the eye.^[28,29]^ In addition, experimental evidence from animal models has confirmed that atropine can also enhance the secretion of dopamine in the retina and increase the thickness of the scleral fibrous layer, both of which can inhibit the development of myopia.^[30,31]^ After comprehensively assessing the therapeutic benefits of atropine for myopia control against its side effects, such as rebound phenomenon upon discontinuation of treatment and photophobia, the concentration of atropine was determined.^[32]^ 0.01% atropine eye drops have become a prevalent therapeutic intervention for the prevention of myopia.^[33]^ Recently, 0.05% atropine has been reported to show superior efficacy in inhibiting myopia compared to 0.01%;^[34]^ more information regarding the long-term therapeutic outcomes of 0.05% atropine is required before it can be validated in clinical practice routinely.

## 5. Conclusion

The present study is the first bibliometric analysis of myopia in children and adolescents. Overall, although the prevalence of myopia in children varies significantly across different areas of the world, an upward trend has been observed in all regions. To address myopia in children and adolescents, the focus of future research funding or international cooperation may be on genetic factors, environmental factors, and intervention factors, including increasing outdoor activity time, using orthokeratology, and controlling with atropine. Our study also serves to identify potential collaborators for researchers with an interest in this field.

Finally, while several compelling findings were obtained through the bibliometric analysis and visualization of publications related to myopia research on children and adolescents, there were also several research limitations to this process, which we further elaborate in accordance with common drawbacks of bibliometric methodologies and our search design constraints.

First, all publications included in our research were exclusively retrieved from the WoSCC database. Incorporating additional widely used databases – such as PubMed, Scopus, and Embase – could potentially improve the comprehensiveness of literature coverage. Moreover, our search strategy only retrieved literature with target keywords appearing in the title or abstract; studies that only mention pediatric or myopia-related terms in keywords or full texts were likely omitted, which may cause incomplete literature retrieval.

Second, the articles analyzed in this study were limited to those published in English, leading to underrepresentation of research literature and authors from non-English-speaking regions.

Third, bibliometric analysis has 2 intrinsic shortcomings. For one thing, it inherently cannot evaluate the methodological quality or risk of bias of individual included studies; a highly cited paper does not equate to a methodologically rigorous study. For another, this analytical method tends to prioritize older publications as newly released articles have insufficient time to accumulate citation counts. This feature may distort the identification of real-time research hotspots and ignore high-impact recent studies.

While these factors may exert some influence on the overall results, their impact on the key trends presented in this paper is minimal. However, in our future research endeavors, we will continue to monitor, track, and further explore emerging research trends within this field. We will also optimize our retrieval strategy by expanding retrieval fields to keywords and full texts and integrating multiple multidisciplinary databases to mitigate the above limitations.

## Acknowledgments

This study was funded by the Independent Research Foundation of the 305 Hospital of PLA (No.25ZZJJLW-004).

## Author contributions

**Conceptualization:** Xiao-Lei Yin, Shu-Xing Ji, Xiu-Xin Li.

**Project administration:** Xiao-Lei Yin.

**Data curation:** Shu-Xing Ji, Xiu-Xin Li.

**Formal analysis:** Shu-Xing Ji, Xiu-Xin Li.
